# Electrical Stimulation-Induced Seizures and Breathing Dysfunction: A Systematic Review of New Insights Into the Epileptogenic and Symptomatogenic Zones

**DOI:** 10.3389/fnhum.2020.617061

**Published:** 2021-01-22

**Authors:** Manuela Ochoa-Urrea, Mojtaba Dayyani, Behnam Sadeghirad, Nitin Tandon, Nuria Lacuey, Samden D. Lhatoo

**Affiliations:** ^1^Department of Neurology, University of Texas Health Sciences Center at Houston, Houston, TX, United States; ^2^Department of Health Research Methods, Evidence, and Impact, McMaster University, Hamilton, ON, Canada

**Keywords:** electrical stimulation-induced seizures, refractory epilepsy, seizure onset zone, epilepsy surgery, outcome, ictal central apnea (ICA), electrical stimulation (ES)

## Abstract

**Objective:** Electrical stimulation (ES) potentially delineates epileptogenic cortex through induction of typical seizures. Although frequently employed, its value for epilepsy surgery remains controversial. Similarly, ES is used to identify symptomatogenic zones, but with greater success and a long-standing evidence base. Recent work points to new seizure symptoms such as ictal central apnea (ICA) that may enhance presurgical hypotheses. The aims of this review are 2-fold: to determine the value of ES-induced seizures (ESIS) in epilepsy surgery and to analyze current evidence on ICA as a new surrogate of symptomatogenic cortex.

**Methods:** Three databases were searched for ESIS. Investigators independently selected studies according to pre-specified criteria. Studies reporting postoperative outcome in patients with ESIS were included in a meta-analysis. For ES-induced apnea, a thorough search was performed and reference list searching was employed.

**Results:** Of 6,314 articles identified for ESIS, 25 were considered eligible to be reviewed in full text. Fourteen studies were included in the qualitative synthesis (1,069 patients); six studies were included in the meta-analysis (530 patients). The meta-analysis showed that favorable outcome is associated with ESIS prior to surgery (OR: 2.02; 95% CI: 1.332–3.08). In addition, the overall estimation of the occurrence of favorable outcome among cases with ESIS is 68.13% (95% CI: 56.62–78.7). On the other hand, recent studies have shown that stimulation of exclusively mesial temporal lobe structures elicits central apnea and represents symptomatogenic anatomic substrates of ICA. This is in variance with traditional teaching that mesial temporal ES is non-symptomatogenic.

**Conclusions:** ES is a tool highly likely to aid in the delineation of the epileptogenic zone, since ESIS is associated with favorable postoperative outcomes (Engel I). There is an urgent need for prospective evaluation of this technique, including effective stimulation parameters and surgical outcomes, that will provide knowledge base for practice. In addition, ES-induced apnea studies suggest that ICA, especially when it is the first or only clinical sign, is an important semiological feature in localizing the symptomatogenic zone to mesial temporal lobe structures, which must be considered in SEEG explorations where this is planned, and in surgical resection strategies.

## Introduction

Electrical stimulation (ES) using invasive electrodes is primarily intended to map eloquent areas of sampled brain in order to formulate resection strategies. In addition, ES can be used to identify seizure onset and symptomatogenic zones (Trébuchon and Chauvel, 2016; Jobst et al., 2020). The seizure onset zone is the “area of cortex from which clinical seizures are generated” (Rosenow and Lüders, 2001). The symptomatogenic zone is the “area of the cortex which when activated by an epileptiform discharge, produces ictal symptoms” (Rosenow and Lüders, 2001). The epileptogenic zone is the area indispensable for the generation of seizures, and may be inferred by defining the symptomatogenic zone, the seizure onset zone, and other areas (Rosenow and Lüders, 2001).

Identification of the seizure onset zone can be challenging in some intracranial EEG studies, and ES can be carried out to reproduce typical seizures, thus lending additional support to identifying putative seizure onset electrodes. However, epilepsy centers differ in their attitudes and approaches to ES (Kovac et al., 2016). There is limited evidence of an association between ES induced seizures (ESIS) and surgical outcomes (Kovac et al., 2016). In addition, stimulation methodology varies between centers, making practice heterogeneous (So and Alwaki, 2018). However, there is some evidence that ESIS have the potential to reliably identify seizure onset zones and help guide successful epilepsy surgery (Munari et al., 1993; Cuello Oderiz et al., 2019; Spilioti et al., 2020; Trebuchon et al., 2021).

Similarly, ES is used to reproduce seizure symptoms, thus identifying symptomatogenic zones (Borchers et al., 2012; Landazuri and Minotti, 2019). Much of this practice is based on long-standing, well-established literature on motor, motor association, sensory, visual, auditory, special sensory, and language cortices (Landazuri and Minotti, 2019). Recent evidence suggests that with appropriate multimodal monioring, semiological signs of breathing disturbances such as ictal central apnea (ICA), reproducible with ES, may enhance presurgical hypotheses for implantation or resection (Lacuey et al., 2017, 2019a).

This review is divided into two parts: (1) a systematic review of literature to determine the state of the art and value of ESIS in epilepsy surgery outcomes, and (2) a review of evidence regarding ICA as a surrogate of symptomatogenic cortex.

## Methods

### ES to Localize the Seizure Onset Zone

Three study authors with expertise in the subject (MOU, NL, and SDL) designed search strategy, terms, and translation in each database. Our main research question was defined as “does removal of seizure onset zone, identified by ESIS, predict favorable surgical outcome in patients with refractory epilepsy?” In addition, we gathered information regarding definitions and stimulation techniques implemented around the world. MEDLINE (via Ovid), EMBASE, and Scopus were searched as of April 1, 2020, without language or date restrictions. The search strategy included the terms “electrical stimulation,” “brain stimulation,” “cortical stimulation,” “epileptogenic zone,” “ictal onset zone,” “seizure onset zone,” and “symptomatogenic zone” (available in Supplementary Material). The search strategy was first developed for MEDLINE and then tailored for each database taking into account differences in vocabulary and syntax rules. Additionally, we incorporated reference list searching (snowballing) into our search strategy. References were exported and managed with Mendeley and duplicates were eliminated. Finally, we adhered to the PRISMA guidelines and checklist to report our findings (Liberati et al., 2009; Moher et al., 2009).

We included randomized/non-randomized controlled trials, cohort studies, and case series reporting use of ESIS in humans, in which methodology of stimulation was described. We included articles in English, Spanish, and French. Unpublished data, studies that did not contain primary data (i.e., review articles), and studies on patients with no diagnosis of epilepsy were excluded. The Rayyan website (Ouzzani et al., 2016) was used for screening of titles and abstracts by three authors (MOU, NL, and SDL), who independently selected studies according to criteria mentioned above. The third reviewer resolved discrepancies in selection between two reviewers. We only included those studies that reported postoperative outcome in patients who had received ES to induce seizures into the meta-analysis.

**Figure 1:**
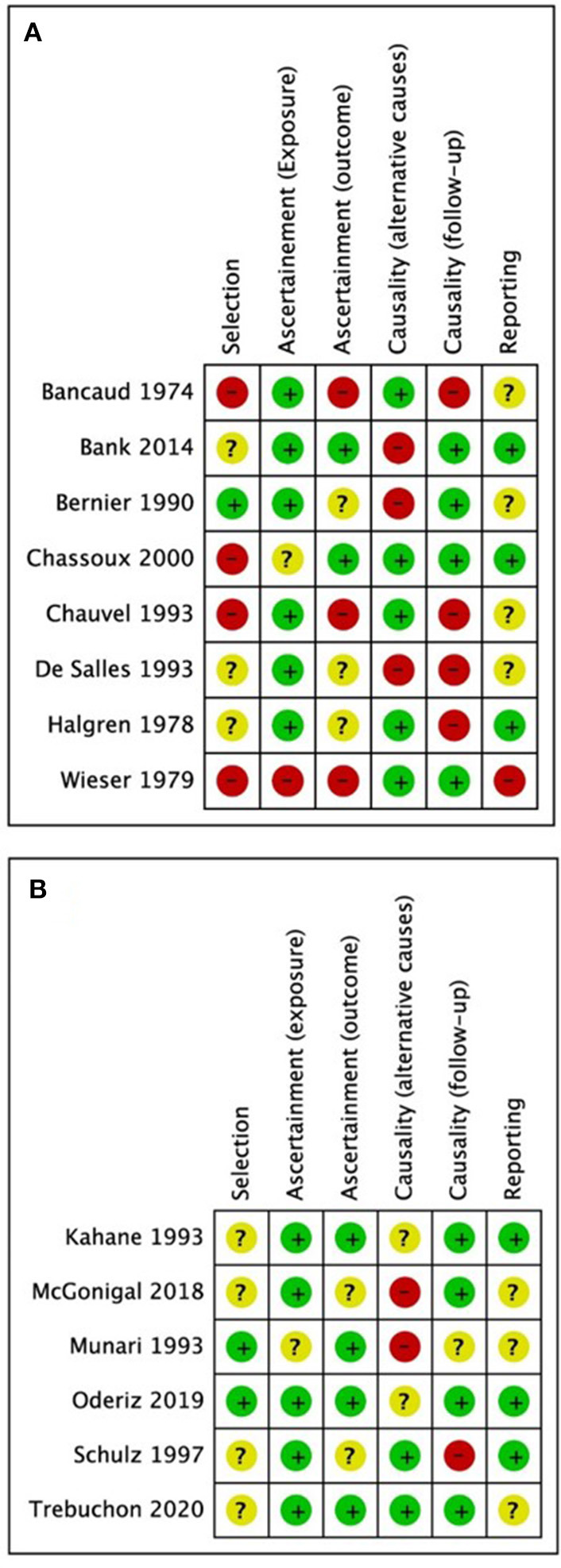
Quality assessment results from **(A)** studies excluded from the meta-analysis; **(B)** studies included in the meta-analysis. Green = low risk of bias, yellow = undetermined risk of bias, red = high risk of bias.

Demographics, stimulation characteristics, rate of induced seizures, outcomes, adverse events, and key conclusions data were collected. Two reviewers (MOU and MD) assessed risk of bias independently and in duplicate using the Cochrane risk of bias instrument (Higgins et al., 2011) with specific adjustments for methodologic evaluation of case series (Murad et al., 2018). The adjusted tool addresses the four following potential sources of bias: selection, ascertainment (exposure and outcome), causality (alternative causes and follow-up), and reporting (Figures 1A,B) (Murad et al., 2018). Any disagreements between reviewers in data extraction or risk of bias assessments were resolved by discussion or involvement of a third reviewer (SDL) as an arbitrator. Using STATA (Version 16.1, College Station, TX), we calculated pooled estimation of rates for favorable outcomes among studies (Engel I). In addition, a conventional meta-analysis with random effect model was used to calculate odds of having favorable outcome following the ES.

### ES for Induction of Central Apnea Semiology

Three study authors with expertise in the subject (MOU, NL, and SDL), designed search strategy and terms to include in a non-systematic literature review. We intended to gather all available information regarding ES and ICA with the goal of summarizing this information. To achieve this we searched MEDLINE (via Ovid) using search terms such as “electrical stimulation,” “cortical stimulation,” “apnea,” and “epilepsy” (for a full description of the search strategy used, please refer to Supplementary Material). This search strategy yielded 140 results. Additionally, we incorporated reference list searching (snowballing) into our search, yielding 157 results. References were exported and managed with Mendeley. An expert in the field of breathing and ES (NL) selected studies that were included in our review. A total of 19 studies were selected that included information regarding apnea as part of the ictal semiology and studies that included ES-induced apnea.

## Results

### ES to Localize the Seizure Onset Zone

**Figure 2:**
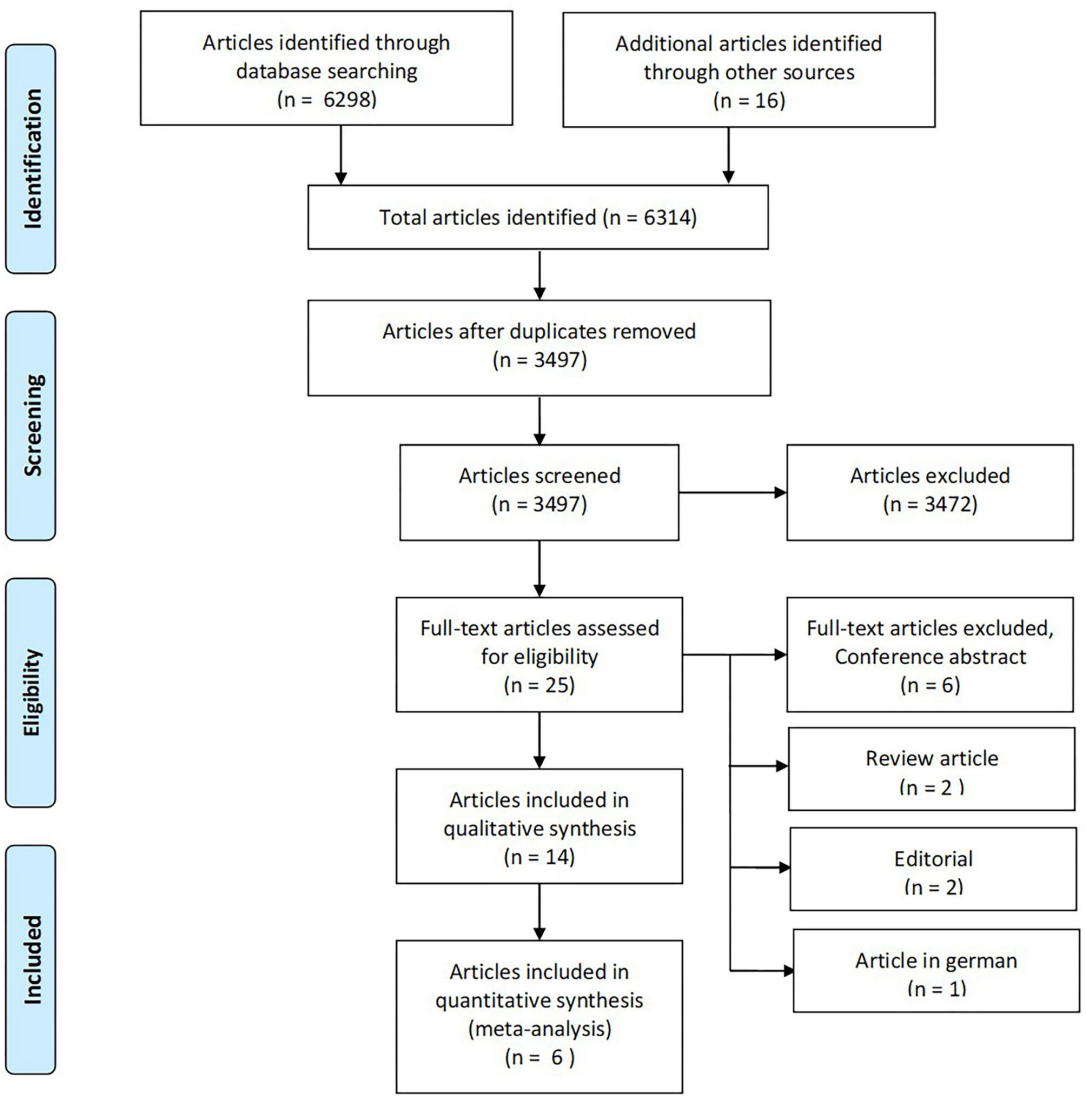
The PRISMA flow diagram for identification of eligible studies for our systematic review and meta-analysis. From Moher et al. (2009).

A total of 6,314 articles were identified through database searches and snowballing (Figure 2). After eliminating duplicates (2,817 duplicates), 3,497 articles were screened using title and abstract. Of these, we excluded 3,472, and 25 were considered eligible for review in full text. Eleven articles were further excluded because they did not meet inclusion criteria, yielding 14 studies included in a qualitative synthesis, comprising 1,069 participants (see Supplementary Table 1 for details of each study). One of the studies retrieved was in abstract form (Trebuchon et al., 2021). Since it was of scientific interest, we contacted the author who provided a full-text article in press. It fulfilled the remaining inclusion criteria, and we decided to include it despite its unpublished status.

The majority of studies were retrospective case series, from France (Bancaud et al., 1974; Wieser et al., 1979; Chauvel et al., 1993; Kahane et al., 1993; Munari et al., 1993; Chassoux et al., 2000; McGonigal et al., 2018), in collaboration with Canada (Cuello Oderiz et al., 2019) and Italy (Trebuchon et al., 2021), and from USA (Halgren et al., 1978; DeSalles et al., 1994; Schulz et al., 1997; Bank et al., 2014) and Canada (Bernier et al., 1990). All were SEEG series, except for three that used subdural grids or strips (DeSalles et al., 1994; Schulz et al., 1997; Bank et al., 2014). For the studies that reported the putative epileptic focus, the most frequent epilepsy type was temporal (56.5%), followed by frontal (25%) and multilobar (11%).

#### Definition of ESIS

We found variability in author definitions of ESIS but broad agreement that it should resemble a spontaneous seizure or aura, described as habitual by the patient or witness (Halgren et al., 1978; Bernier et al., 1990; Kahane et al., 1993; Munari et al., 1993; Cuello Oderiz et al., 2019; Trebuchon et al., 2021). Munari et al. defined it as a clinical manifestation, either subjective or objective, resembling those previously described by patient and/or witnesses or as seen during seizure recordings (Munari et al., 1993). Bernier et al. added the electrophysiological aspect, defining it as first signs of a seizure resembling the patient's habitual symptoms in the context of a simultaneous after discharge localized to the stimulation contacts only (Bernier et al., 1990). Similarly, for other authors, electrographic changes with spatial and temporal evolution had to accompany clinical changes (Bank et al., 2014; Cuello Oderiz et al., 2019). In contrast, some specified absence of ES-induced afterdischarges as a main characteristic of stimulation-induced aura (Schulz et al., 1997).

#### Stimulation Parameters and Responses

Both low-frequency (<5 Hz) and high-frequency (>5 Hz) stimulation approaches were used. High-frequency stimulation was used in all included studies at 50 Hz, with one exception where stimulation frequency was not specified (Wieser et al., 1979). One study also used 10- and 20-Hz stimulation (Bank et al., 2014). Pulsewidth varied from 0.1 to 3 ms (Supplementary Table 1). High-frequency stimulation was usually 50-Hz pulses using 0.2–1 ms pulse width. When low-frequency stimulation at 1 Hz was used, the train duration lasted from 20 to 40 s (Kahane et al., 1993; Munari et al., 1993; Cuello Oderiz et al., 2019). On the other hand, high-frequency stimulation was carried out from 3 to 10 s (Bancaud et al., 1974; Bernier et al., 1990; Chauvel et al., 1993; Kahane et al., 1993; Munari et al., 1993; Schulz et al., 1997; Bank et al., 2014; McGonigal et al., 2018; Cuello Oderiz et al., 2019). Current intensity varied between studies. A titrated approach was used starting with low intensities of 0.25–0.5 mA, increasing progressively to a maximum of 15 mA, with lower intensities used in temporal lobes (Cuello Oderiz et al., 2019).

ESIS were elicited on average in 69.4% ± 23.6 (range 27.7–100) of patients (Halgren et al., 1978; Bernier et al., 1990; Chauvel et al., 1993; Munari et al., 1993; DeSalles et al., 1994; Schulz et al., 1997; Bank et al., 2014; McGonigal et al., 2018; Cuello Oderiz et al., 2019; Trebuchon et al., 2021). These rates varied depending on type of stimulation. Cuello Oderiz et al. found high-frequency stimulation (50 Hz) to be more effective in inducing seizures (54.9 vs. 18.2%) (Cuello Oderiz et al., 2019). Similarly, Kahane et al. reported 37.5% seizures with high frequency vs. 9.7% with low frequency (Kahane et al., 1993). ES-induced adverse effects were more frequent with high frequency (5.9 vs. 3.5%) compared to low frequency (Munari et al., 1993). Adverse effect rates up to 10% rate were found with high-frequency settings in one study (Kahane et al., 1993). Difficulty with mouth opening and restricted cephalic symptoms were adverse effects of ES (Munari et al., 1993; DeSalles et al., 1994). In addition, atypical electroclinical seizures, although rare, were reported to be more frequent with 50-Hz stimulation in one study (7.8 vs. 1.5%) (Cuello Oderiz et al., 2019). Rate and type of adverse effects were not described in some studies (Bancaud et al., 1974; Wieser et al., 1979; Bernier et al., 1990; Chauvel et al., 1993; Schulz et al., 1997).

#### Meta-Analysis: Epilepsy Surgery Outcome

**Figure 3:**
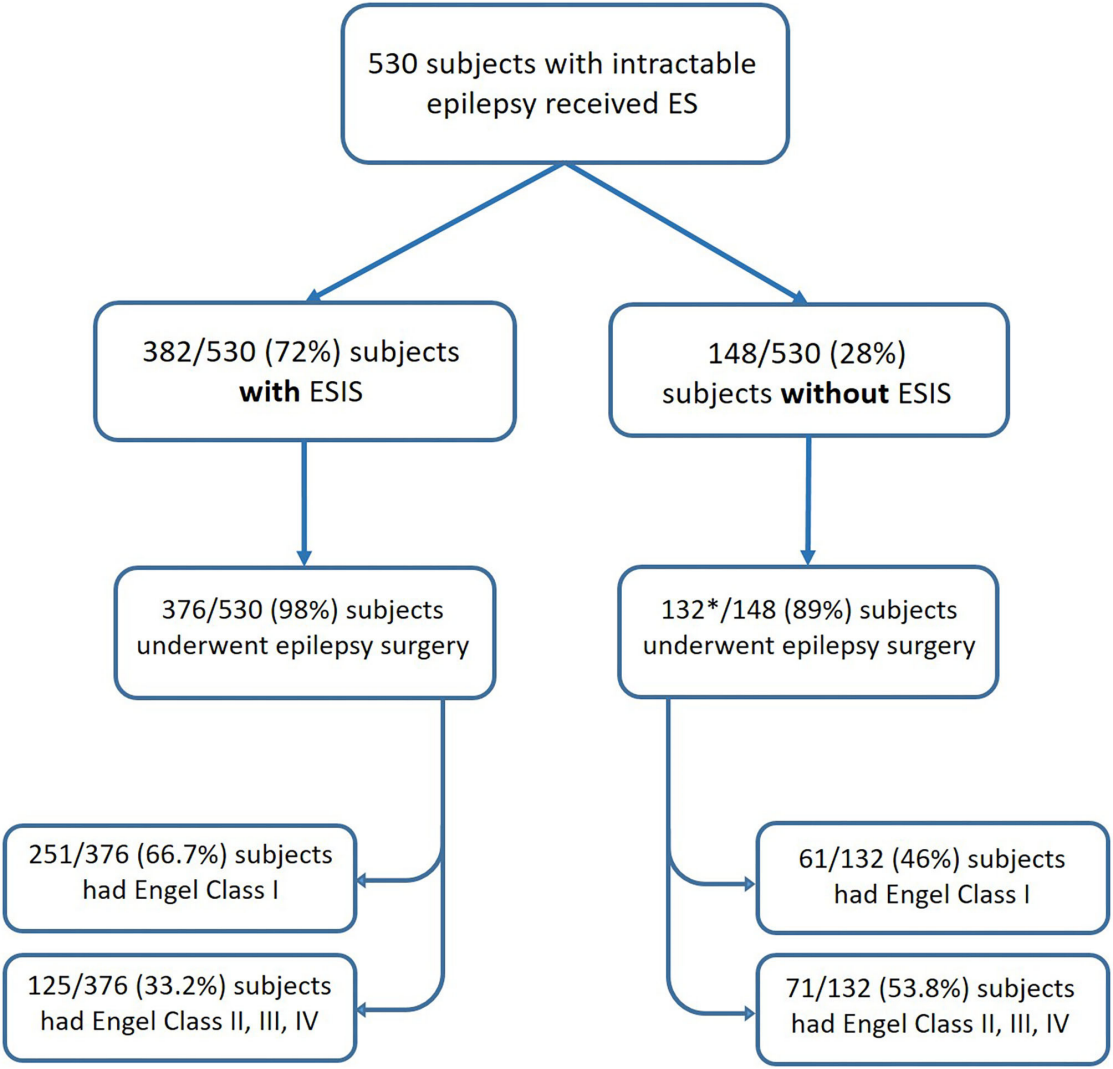
Flowchart showing the number of patients categorized by status of ES and their post-surgical outcomes. *One study included in the meta-analysis did not report the number of patients without ESIS who underwent epilepsy surgery (Schulz et al., 1997). ES, electrical stimulation; ESIS, electrical stimulation-induced seizures.

We included in our meta-analysis studies that reported outcomes after epilepsy surgery. Only six studies fulfilled criteria (Kahane et al., 1993; Munari et al., 1993; Schulz et al., 1997; McGonigal et al., 2018; Cuello Oderiz et al., 2019; Trebuchon et al., 2021). The sample sizes of individual studies included ranged from 10 to 346. In total, included studies consisted of 530 subjects with intractable epilepsy who received ES, of which a total of 508 underwent epilepsy surgery with reported outcomes. Among those who underwent surgery, 376 cases had ESIS, and 132 did not have ESIS (Figure 3).

**Figure 4:**
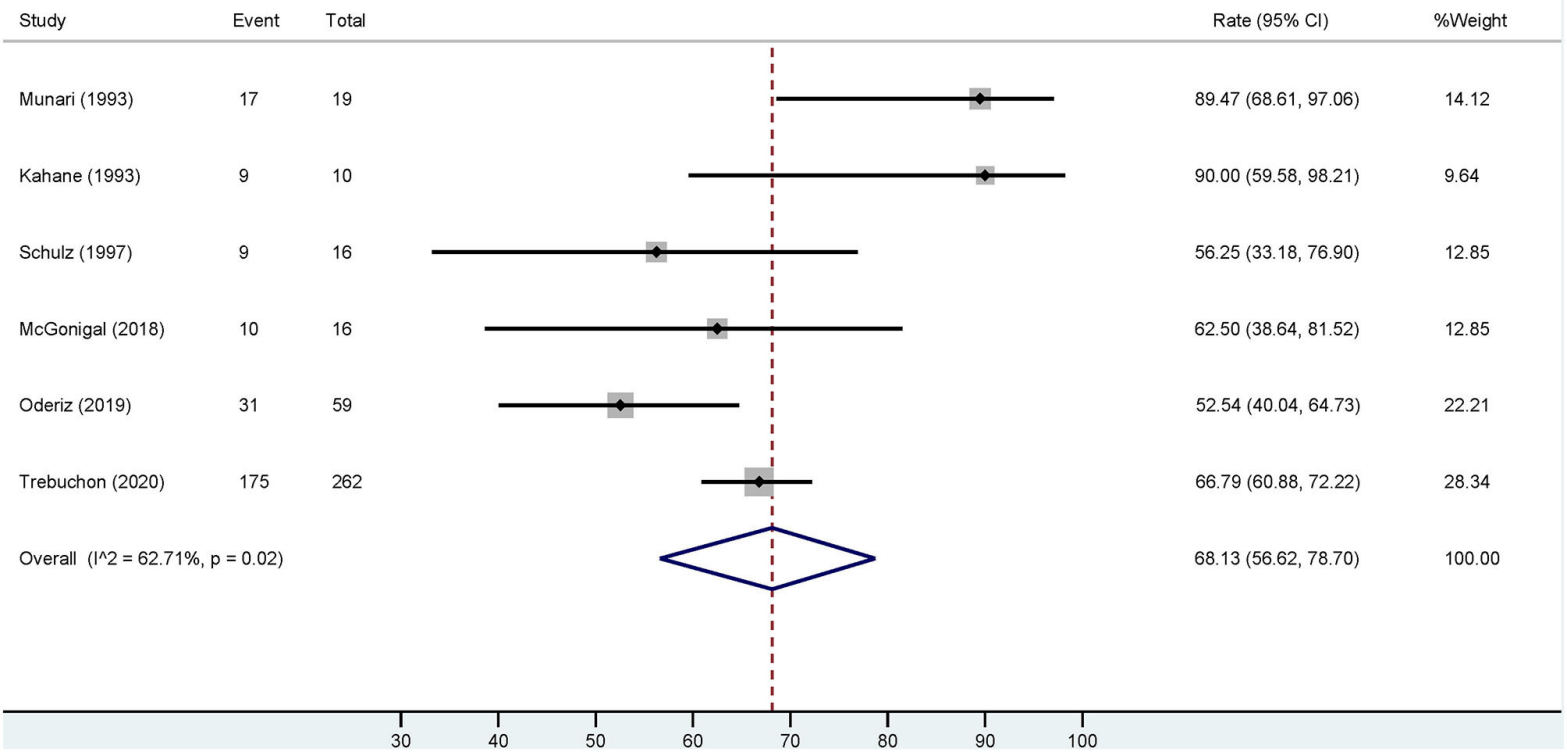
Forest plot depicting the pooled estimate of favorable outcomes after epilepsy surgery in patients with ESIS. “Event” indicates the number of patients who had a favorable outcome and “Total” indicates the number of patients who had ESIS. Rates of favorable outcome for each study are seen to the right of the figure with their respective weights. The red dashed line indicates the pooled estimated favorable outcome value. The blue diamond indicates the pooled confidence interval. Each study is represented by a black line that indicates the confidence interval, a square that represents the weight of each study, and a dot indicating the rate of favorable outcome. The studies are heterogeneous as demonstrated by an *I*^2^ value of 62.7%.

Based on the six studies included, our meta-analysis revealed that overall estimation of the occurrence of favorable outcome (Engel class I) among cases who had ESIS is 68.13% [95% confidence interval (CI): 56.62–78.7] (Figure 4). After excluding the study with the highest event rate of a favorable outcome (Trebuchon et al., 2021), the overall estimated rate did not change.

Four studies investigated epilepsy surgery outcome in patients with ESIS (Munari et al., 1993; Schulz et al., 1997; Cuello Oderiz et al., 2019; Trebuchon et al., 2021). Schulz et al. analyzed if ES-induced auras, without afterdischarges or seizures, could delineate a resectable epileptogenic zone (Schulz et al., 1997). Complete resection of the ESIS demarcated area did not correlate with favorable postoperative outcomes (Schulz et al., 1997). In contrast, Cuello Oderiz et al. found ESIS to portend favorable surgical outcomes (Cuello Oderiz et al., 2019). In the favorable outcome group, the percentage of ESIS was greater and the number of resected electrode contacts where stimulation induced seizures was higher, compared to the poor outcome group. They concluded that ESIS could be an indicator of the epileptogenic zone. In line with these findings, Trebuchon et al. indicated, in a multivariate analysis of 346 patients, that ESIS with low-frequency parameters was independently associated with positive seizure outcome after surgery (Trebuchon et al., 2021). In addition, Munari et al. reported that patients in whom low-frequency stimulation was effective in inducing seizures and who underwent epilepsy surgery had a favorable outcome (Engel I) with a mean follow-up of 15.4 months (Munari et al., 1993).

**Figure 5:**
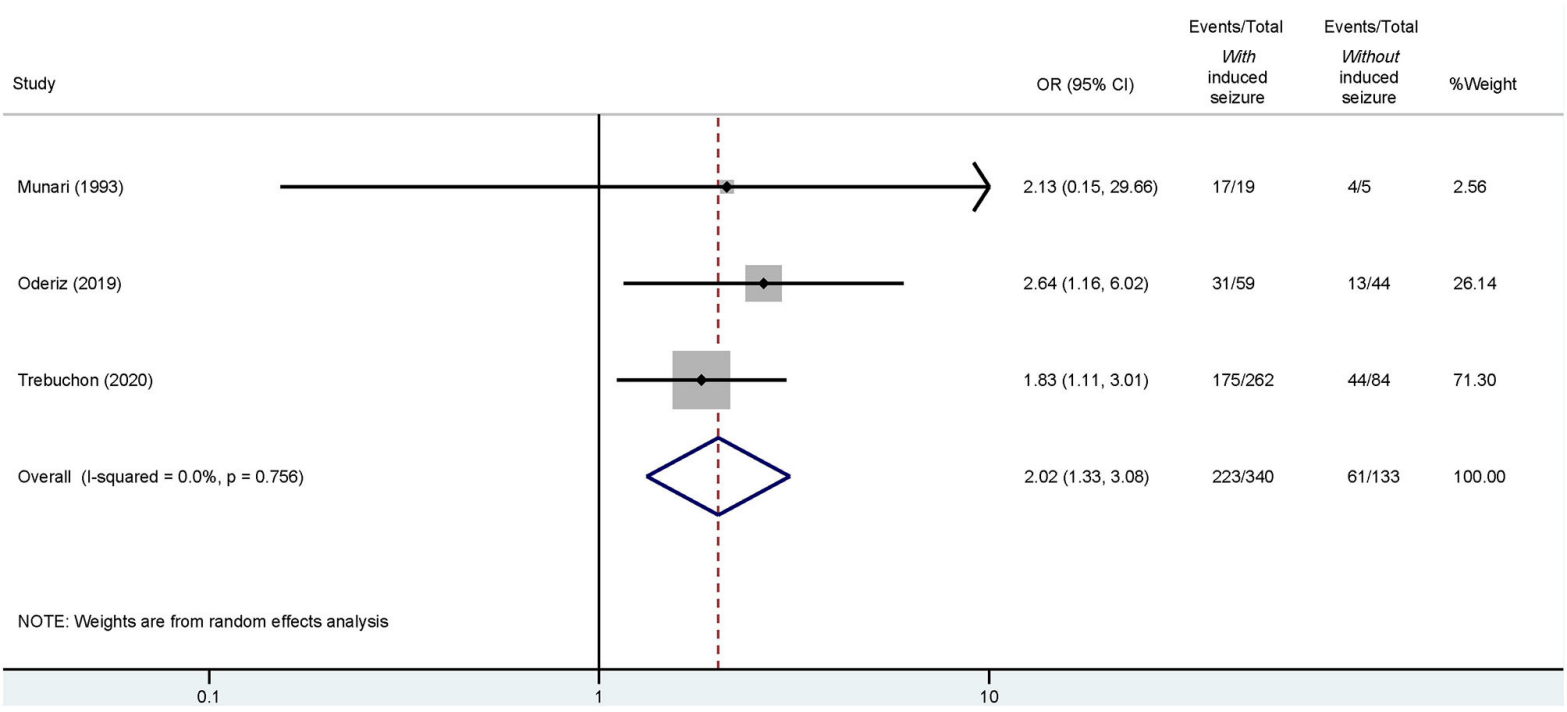
Forest plot elucidating the association between ESIS and favorable outcome after epilepsy surgery. “Events/Total” reflects the number of patients with favorable outcome who underwent epilepsy surgery with or without ESIS over the total of patients with or without ESIS. Overall odds ratio (OR) is indicated by the red dashed line. The diamond indicates the pooled confidence interval. The black lines represent the confidence intervals of each study with the square depicting the weight of each study and the dot pointing at the OR of each study.

Of these four studies, three reported detailed outcomes based on favorable (Engel I) and unfavorable (Engel II–IV) results among those with/without ESIS (Munari et al., 1993; Cuello Oderiz et al., 2019; Trebuchon et al., 2021). Thus, we conducted another conventional meta-analysis with these studies to assess a possible association between favorable outcomes and ESIS. Results showed that favorable outcome is associated with presence of ESIS with an odds ratio (OR) of 2.02 (95% CI: 1.33–3.08) compared to its absence (Figure 5).

### ES for Induction of Central Apnea Semiology

Whereas ES is widely used for identification of functional cortex, ES can also be used to reproduce seizure signs and symptoms (Rasmussen and Penfield, 1947; Penfield and Perot, 1963; Borchers et al., 2012; Landazuri and Minotti, 2019). Identification of cortical areas involved in symptom production is important to understand the epileptogenic zone and seizure propagation networks. ICA has been recently identified as a semiological sign in temporal lobe epilepsies (Lacuey et al., 2019a), which is elicitable and may be of value in seizure analysis for presurgical assessments.

#### Historical Perspective

Previous evidence in animals suggests that respiratory arrest can be induced by ES of temporal pole, uncus, insula, rostral cingulate, and posterior orbitofrontal cortex (Bailey and Sweet, 1940; Smith, 1945; Kaada et al., 1949; Hoffman and Rasmussen, 1953). These studies found ES to predominantly affect the inspiratory phase (Smith, 1945; Delgado and Livingston, 1948; Kaada et al., 1949). Breathing arrest continued during the stimulation period, with resumption of normal breathing pattern once stimulation ceased. Simultaneous with apnea, other responses were variably recorded such as blood pressure and heart rate changes, decrease in gastric tone, and vocalization (Bailey and Sweet, 1940; Smith, 1945; Hoffman and Rasmussen, 1953). These studies were done under the effect of general anesthesia (Kaada et al., 1949).

Similar breathing responses were found in humans. In 1899, Hughlings Jackson described what he called uncinated fits, seizures whose semiology included respiratory arrest (Jackson, 1899). On stimulation of posterolateral orbital cortex and rostral cingulate in patients with psychiatric conditions undergoing intracranial procedures such as frontal lobotomies, scientists noted complete cessation or decrease in breathing lasting the stimulation period (Chapman et al., 1949; Pool and Ransohoff, 1949). Later, Penfield's group found better demarcation of regions, which, when stimulated in the conscious patient with local anesthesia, elicited arrest of breathing in the same regions as in monkeys (Kaada and Jasper, 1952). Specifically, this study found respiratory arrest with stimulation of the anterior hippocampal gyrus, temporal pole (medial and ventral), anterior insula, and anterior cingulate gyrus. Similarly to previous studies, respiratory arrest occurred with the chest assuming an expiratory position and was occasionally interrupted by a deep breath (Kaada and Jasper, 1952). Nelson and Ray reproduced these findings in one patient by stimulating the amygdala (Nelson and Ray, 1968). One caveat of these studies was likely imprecise localization of cortical areas stimulated, given the technical limitations of the period.

#### The Present of ES-Induced Central Apnea

In the last decade, the use of intracranial EEG studies with simultaneous multimodal polygraphy in the Epilepsy Monitoring Unit (EMU) has allowed for the investigation of important ictal and peri-ictal signs such as hypoxemia, central and obstructive apnea, laryngospasm, and hypercarbia (Kennedy and Seyal, 2015; Esmaeili et al., 2018; Lacuey et al., 2018a,b,c, 2019a; Murugesan et al., 2019; Vilella et al., 2019a,b; Allen et al., 2020). Pulse-oximetry, respiratory inductance plethysmography, nasal/oral thermistors and pressure transducers, capnography, and transcutaneous CO_2_ sensors are available and provide valuable information for better understanding of breathing (Dlouhy et al., 2015). However, their use is not widespread and mainly used by SUDEP researchers.

Using ES of intracranial electrodes in patients admitted to EMU as a prelude to epilepsy surgery, several brain areas have been identified as regions where ES induces central apnea: amygdala, hippocampal head and body, anterior parahippocampal, and antero-mesial fusiform gyri (Dlouhy et al., 2015; Lacuey et al., 2017, 2019b; Nobis et al., 2018). These regions represent symptomatogenic anatomical substrates of ICA (Lacuey et al., 2019a). Contrary to studies in animals and historical stimulations in humans, with recent, anatomically precise SEEG guided stimulations, no breathing responses have been elicited from lateral temporal or orbitofrontal neocortex (Lacuey et al., 2017). As in animals, ICA occurs with the thorax adopting a resting position, allowing expiration to occur but not inspiration (Lacuey et al., 2019b). Apnea has been elicited in both hemispheres in all studies (Dlouhy et al., 2015; Lacuey et al., 2017, 2019b; Nobis et al., 2018). A minority of patients exhibit hypopnea during stimulation (Nobis et al., 2018). Interestingly, patients reported no symptoms during stimulation (apnea agnosia) and breathing cessation could be overridden if the patient was asked to voluntarily breathe (Lacuey et al., 2017; Nobis et al., 2018). Apneic responses also occurred during sleep (Nobis et al., 2018). At times, apnea lasted less than the stimulation period. In others, it continued after stimulation had stopped. Duration of apnea correlated with duration of stimulation (Lacuey et al., 2019b).

Parameters used for ES of breathing changes are similar to those used for cortical mapping (1, 5, 20, and 50 Hz frequencies; 0.2 pulse width; 2 to 40 s train duration; current intensity up to 10 mA; and 20 V) (Dlouhy et al., 2015; Lacuey et al., 2017, 2019b; Nobis et al., 2018). To monitor for breathing, researchers have simultaneously used inductance plethysmography to record chest and abdomen excursions, nasal thermistors to measure nasal airflow, capnographs for end tidal CO_2_, or digital transcutaneous CO_2_ sensors and electrocardiogram, along with EEG. One study also monitored beat-to-beat arterial blood pressure during stimulation sessions using continuous noninvasive arterial pressure monitoring (Lacuey et al., 2019b). The higher the stimulus current and the longer the stimulation, the greater the duration of ICA (Lacuey et al., 2019b). Frequency of stimulation affects apnea latency with low frequencies (1 Hz) provoking delayed apnea (apnea began 1–2 breaths after stimulation onset) and high frequency (50 Hz) inducing immediate breathing arrest (Lacuey et al., 2019b).

Of note, the definition of ICA has varied between studies. These include one (Vilella et al., 2019a) or two involuntary missed breaths (Lacuey et al., 2017), and an abrupt drop in peak signal excursion by >90% of pre-event baseline, cessation of breathing lasting for at least 6 or ≥10 s (Lacuey et al., 2018c, 2019a,b), or respiratory arrest for the duration of the ES (Nobis et al., 2018).

#### ICA as a Semiological Phenomenon

Studies indicate that ICA is a semiological phenomenon, hypothesized to arise from seizure activity in symptomatogenic brain regions, including mesial temporal structures (Lacuey et al., 2017; Vilella et al., 2019a). Recent investigations have shown that ictal activity that spreads to the amygdala elicits ICA (Nobis et al., 2020; Rhone et al., 2020). This finding supports previous observations that report that ICA preceded surface electroencephalographic onset of temporal lobe seizures, representing the first sign in 69% and occasionally the only clinical seizure manifestation (in 16% of the seizures) (Nadkarni et al., 2012; Lacuey et al., 2019a). ICA is associated with significant oxygen desaturation, especially if prolonged, and with longer EEG recovery duration (time from EEG seizure end to continuous slow generalized EEG activity) (Lacuey et al., 2018c, 2019a; Vilella et al., 2019a). ICA occurs in 36.5–44% of focal onset seizures, with a higher incidence (68.7%) in mesial temporal lobe compared to extratemporal epilepsy (OR: 10.1, 95% CI: 5.5–18.5; *p* = 0.001) (Bateman et al., 2008; Lacuey et al., 2018c, 2019a; Vilella et al., 2019a). It is exclusive to focal epilepsy (Lacuey et al., 2018c). As such, ICA as an isolated sign may indicate a mesial temporal lobe epilepsy and potentially be used as a localizing sign in presurgical epilepsy evaluation (Lacuey et al., 2018c, 2019a).

## Discussion

### ESIS: The Seizure Onset Zone

The present systematic review of literature addresses ESIS as a technique to identify the seizure onset zone and improve outcome in epilepsy surgery. Our meta-analysis demonstrated that ESIS is associated to a greater likelihood of favorable surgical outcomes and can be used to adequately determine the seizure onset zone. We found that the definition of ESIS is heterogeneous and that ES parameters vary from study to study.

In our meta-analysis, patients with ESIS were twice as likely to have favorable outcome compared to those without ESIS. Contrary to previous studies showing discrepancies in apparent benefit in postsurgical outcomes (Kovac et al., 2016), our meta-analysis shows that ESIS are associated with favorable outcome (Engel I) after epilepsy surgery. Our meta-analysis, based on heterogeneous studies with a reasonably low/intermediate risk of bias, allows us to conclude that it may be a valuable aid to epilepsy surgery resection planning. We confirmed this even after excluding the study with the highest event rate of a favorable outcome.

Literature indicates that ES for seizure induction is a technique predominantly practiced in Europe, and less used in North America despite it being a technique employed for more than five decades. The difference in practice between countries may be explained by history and traditions. Europe has been greatly influenced by Bancaud and Tailarach's work in which induction of seizures was part of routine invasive study and helped define the epileptogenic zone (Kovac et al., 2016). On the other hand, North America follows Penfield's influence who emphasized in using ES in the operating room for eliciting afterdischarges and seizures to identify the seizure focus (Jasper, 1954). Although Penfield advocated for the use of ESIS, this practice did not become widespread in countries like the United States.

Strikingly, definition of a reliable ESIS varies greatly between studies. All authors agree in that ESIS must reproduce symptoms and signs of habitual seizures that the patient or a witness has described. Some authors take into account the electroclinical correlation of the events (Bernier et al., 1990; Bank et al., 2014; Cuello Oderiz et al., 2019); some only mention a clinical definition (Kahane et al., 1993; Munari et al., 1993; Schulz et al., 1997; Trebuchon et al., 2021). This variability affects reproducibility and homogeneity of investigational conclusions. In addition, definitions using exclusively clinical features could potentially erroneously localize symptomatogenic zones instead of seizure onset zone (Velez-Ruiz, 2019). For these reasons, we concluded that a unified definition of reliable ESIS should be stated and used in future research. Having a unified definition will decrease difficulties that arise when comparing studies, allow for better reproducibility of results, and increase external validity of present and future research. In this regard, Cuello Oderiz et al.'s definition is comprehensive and applicable where a typical ESIS is “a seizure with clinical semiology resembling spontaneous seizure or aura and required to have electrographic changes with clear spatial and temporal evolution” (Cuello Oderiz et al., 2019). Additionally, if spontaneous seizures have been previously captured during invasive evaluation, electrographic similarity between spontaneous and induced seizures is essential.

Both low- and high-frequency ES have been employed in the studies reviewed. Induction of ESIS occurs in 51.6–75.3% of stimulated patients (Schulz et al., 1997; Cuello Oderiz et al., 2019; Trebuchon et al., 2021). Although high-frequency stimulation appears more effective in inducing seizures, it is also associated with greater frequency of adverse effects and false positives (Munari et al., 1993). False positives should be recognized as clinical events that are not acknowledged by the patient as typical, nor they have been observed spontaneously before in that patient, and they are not related to the anatomical site of ES (Munari et al., 1993). ES should be performed with the patient fully medicated so as to avoid induction of atypical seizures (Engel and Crandall, 1983; Cuello Oderiz et al., 2019). For these reasons, and since 50-Hz stimulation artifact obscures electrographic seizure onset, in our center, stimulation is started at 1 Hz and, if no seizures are elicited after increasing current intensity progressively, frequency parameters are progressively increased to 5, 20, and 50 Hz sequentially. Of note, the mesial temporal lobe is sensitive to low-frequency stimulation, and high-frequency stimulation is prone to inducing unwanted generalized convulsive seizures (Munari et al., 1993).

Comparison between ESIS and spontaneous seizures regarding epilepsy surgery and its outcome has been undertaken (Cuello Oderiz et al., 2019) and suggests that ESIS may potentially replace spontaneous seizures during presurgical invasive evaluation (Cuello Oderiz et al., 2019). Further attempts have been made to determine comparability of ESIS to spontaneous seizures. Spilioti et al. reported their experience with induced seizures during ES when mapping eloquent cortex (Spilioti et al., 2020). They elicited seizures in 23.4% of patients, of whom 55.9% where typical (similar to spontaneous seizures) and 41% were atypical. In 73.7% of the patients, the contacts involved in ESIS were fully concordant with the contacts involved in spontaneous seizures registered *a priori* (Spilioti et al., 2020). This suggests that ESIS may be a surrogate for spontaneous seizures during intracranial EEG. This approach may reduce the time needed for invasive EEG and may spare the need for decreasing anti-seizure medication since ES is performed under the effect of the patient's habitual treatment. However, the concordance between ESIS and spontaneous seizures needs further research. Of the studies included in the systematic review, none addressed this subject in a systematic manner.

Since the majority of studies assessed in this systematic review are retrospective, there is an urgent need for prospective randomized control trials that further elucidate the value of ESIS, making further comparisons with spontaneous seizures and providing additional information regarding effective, adverse event-free stimulation parameters.

### ESIS Signs and Symptoms: Central Apnea and the Symptomatogenic Zone

Mesial temporal structures have not routinely stimulated for functional mapping since traditionally teaching holds these structures to be “silent” to ES (Spilioti et al., 2020). However, we find recent evidence to suggest that with appropriate multi-modal monitoring, these structures are highly symptomatogenic in both spontaneous seizures and ES, thus opening up a whole new aspect to epilepsy surgery assessments. From ES studies in animals and humans, we know that mesial temporal structures have a modulatory effect on breathing (Bailey and Sweet, 1940; Kaada et al., 1949; Kaada and Jasper, 1952; Dlouhy et al., 2015; Nobis et al., 2018; Lacuey et al., 2019b; Rhone et al., 2020). The mechanism underlying ICA is due to seizure discharge induced impairment of involuntary, suprapontine (amygdalohippocampal) breathing control through disruption of brainstem-driven inspiration (Lacuey et al., 2018c). Of the several limbic and paralimbic structures identified, amygdala influences appear most consistent. Connections of the amygdala, especially its mesial and basal regions, with brainstem respiratory nuclei allow for better understanding of breathing disruption during seizures that involve this structure, but also links amygdala and temporal mesial structures to SUDEP pathophysiology (Manolis et al., 2019). Apnea agnosia is an intriguing aspect and may reflect seizure-induced inhibitory influences that prevent chemosensitivity-driven apnea awareness. It also explains why apnea has gone unrecognized in epilepsy semiological analysis for so long. With increasing use of multimodal cardio-respiratory polygraphy, this is likely to change.

From the studies analyzed, we find that ES-induced ICA/hypopnea is an understudied but a potentially valuable localizing sign for epilepsy surgery implantation and/or resection. Central apnea is a likely result of seizure activity (ICA) and may be reproduced by ES of the mesial temporal structures, especially amygdala. One of the pillars of successful epilepsy surgery and resection hypotheses includes detailed understanding of the symptomatogenic zone with which to plan a surgery. Our review of the recent literature allows us to conclude that ES-induced ICA is a relevant sign when localizing the symptomatogenic zone for epilepsy surgery. In seizure semiology chronology, ICA appearance may be the first and at times only semiological phenomenon. This potentially allows delineation of the patient's symptomatogenic and ictal onset zones and, consequently, the putative epileptogenic zone (Lacuey et al., 2019a). Any planned SEEG exploration then must include the brain areas known to generate ICA, including amygdala, hippocampus, anterior parahippocampal, and antero-mesial fusiform gyri. Therefore, since ICA is a reliable sign of mesial temporal lobe seizures, efforts to record breathing disturbances in the epilepsy monitoring unit should be made, with systematic monitoring using respiratory belts. Where ICA is a later semiological component in the patient's seizure chronology, implantation of the same substrate structures may still throw light on seizure spread and spread pathways.

### Limitations

Our study has several limitations. Firstly, the number of studies in literature that have addressed ESIS is limited. We found only 14 studies, of variable quality, and of those, less than half could be included in the meta-analysis. In addition, studies are heterogeneous and definitions, stimulation parameters, and outcomes vary from study to study. We did not find any randomized controlled study from which to draw high-quality information. The studies pooled in the meta-analysis were of intermediate quality, and this allowed us to draw some conclusions that have been presented.

## Conclusions

ESIS constitute a reliable technique to delineate the epileptogenic zone and provide distinct and complementary information to guide epilepsy surgery. Prospective research addressing ESIS is urgently needed, with special emphasis on efficacy and safety using the definition proposed in this review. This will provide the necessary evidence base, protocols, and guidelines for usage of this important aspect of patient assessment. ES of brain structures (amygdala, hippocampal head and body, anterior parahippocampal, and antero-mesial fusiform gyri) reliably demonstrated to produce central apnea suggests that these brain regions likely generate ICA. Therefore, when ICA is a seizure semiology component, these areas should be included in the surgical hypothesis, and SEEG exploration strategy if this is planned. ICA is particularly valuable if it is the first or only semiological sign, but because of apnea agnosia, this sign can only be identified if appropriate respiratory monitoring is employed in the EMU.

## Data Availability Statement

The original contributions presented in the study are included in the article/Supplementary Material, further inquiries can be directed to the corresponding author.

## Author Contributions

SL conceived the presented idea. MO-U, NL, and SL completed the systematic review. MD and BS performed the meta-analysis and did the figures. MO-U wrote the manuscript with support from NL, SL, and NT. All authors contributed to the article and approved the submitted version.

## Conflict of Interest

The authors declare that the research was conducted in the absence of any commercial or financial relationships that could be construed as a potential conflict of interest.
