# Supplementary material for: Electrical Stimulation-Induced Seizures and Breathing Dysfunction: A Systematic Review of New Insights Into the Epileptogenic and Symptomatogenic Zones
Source: Front Hum Neurosci. 2021 Jan 22;14:617061. doi: 10.3389/fnhum.2020.617061 (PMC7862564; doi:10.3389/fnhum.2020.617061)
Supplement: Supplementary file 1 [file Table_1.docx]

**Table 1.** Summary of included studies. R = retrospective; P = prospective; F= female; EO = extra-operative; IO = intra-operative; N = number of participants; NR = not reported; ESIS = electrical stimulation induced seizures; LFS = low frequency stimulation; HFS = high frequency stimulation; Fro= frontal epilepsy; Post= posterior epilepsy; T= temporal epilepsy; C= central epilepsy; ML= multilobar epilepsy; FU= follow-up; ms= milliseconds; s= seconds

|  |  | |  |  |  |  |  |  | **Epilepsy localization** | | | | | **Stimulation parameters** | | | |  | |  |  |  |
| --- | --- | --- | --- | --- | --- | --- | --- | --- | --- | --- | --- | --- | --- | --- | --- | --- | --- | --- | --- | --- | --- | --- |
| **Study** | **Country** | **Type** | | **N** | **F %** | **Mean age** | **Set-ting** | **Tech-nique** | **Fro** | **Post** | **T** | **C** | **ML** | **Freq** | **Pulse width (ms)** | **Duration (s)** | **Intensity (mA)** | **Patients with ESIS** | **POP outcome in ESIS group** | | **Adverse events of ES** | **FU (months)** |
| Cuello Oderiz C, et al. JAMA Neurol. **2019**;76(9):1070–8 | Canada, France | R | | 103 | 52 | 31 | EO | SEEG | 27 | 3 | 27 | 0 | 39 | 1H 50HZ | 0.5-1.5 0.5-1 | 20-40 5 | 0.5 to 5 in temporal, 8 in neocortex | 59 (57.3%) | | 42.7%  Engel class I | None | median 42.2 m (12-122.1) |
| Chauvel P, et al. Adv Neurol. **1993**;63:115–21 | France | | R | 72 | NR | 25 | EO | SEEG | 28 | 0 | 44 | 0 | 0 | 1 Hz 50Hz | 0.1-1 1 | 5 | 0.5-5 | 38 (52.8%) | | NR | NR | NR |
| Bank AM, et al. Epilepsy Behav. **2014**;34:6–8. | USA | | R | 57 | 53 | 30.5 | EO | Sub-dural grids | NR | NR | NR | NR | NR | 10, 20, 50 Hz, | 0.2-0.5 | 5 | 8.0-15 mA | 19 (33%) | | NR | Disruption of mapping | 12 to 24 m |
| Schulz R, et al. Epilepsia. **1997**;38(12):1321–9. | USA | | R | 31 | NR | 25.1 | EO | Sub-dural grids | 9 | 3 | 3 | 0 | 0 | 50 Hz | 0.3 | 5 to 10 | 15 max | 16 (51.6%) auras | | NR | NR | mean of 15.7 m |
| McGonigal A, et al. Epilepsy Behav. **2018**;88:262–7. | France | | R | 16 | 56 | 26.5 | EO | SEEG | 11 | 0 | 6 | 4 | 0 | 50 Hz | 1 | 3 to 5 | 0.5-2 mA | 16 (100%) | | 76.9% Engel I | None | more than 12 m |
| Munari C, et al. Acta neurochirurgica.**1993.** p. 181–5. | France | | P | 24 | 58 | 25.2 | EO | SEEG | 0 | 0 | 16 | 0 | 0 | 1Hz 50Hz | 3 1 | 40 5-7 | 0.25-4 mA | 19 (79%) | | 100% Engel I | 3.5% with LFS and 5.9% HFS= localized cephalic symptoms | mean of 15.4 m |
| Bernier GP, et al. Epilepsia. **1990**;31(5):513–20 | Canada | | R | 126 | NR | NR | EO | SEEG | 21 | 8 | 51 | 0 | 33 | 50 Hz | 0.5 | 5 | 10 mA | 79 (62.7%) | | NR | NR | NR |
| DeSalles A, et al. Proc XIth Meet World Soc Stereotact Funct Neurosurg. **1994**;62:226–31 | USA | | P | 8 | 0 | 40 | EO | Sub-dural strips | NR | NR | NR | NR | NR | 60Hz | 0.3 | NR | 15 mA | 6 (75%) | | 94% had >75% reduction in  seizures | 1 mouth opening limitation | NR |
| Wieser HG, et al. Epilepsia. **1979**;20(1):47–59. | France | | R | 202 | NR | NR | EO | SEEG | 0 | 0 | 202 | 0 | 0 | NR | NR | NR | NR | 56 (68%) | | NR | NR | 60 m |
| Kahane P, et al. Neurophysiol Clin  **1993**;23(4):305–26. | France | | P | 10 | 60 | 22.9 | EO | SEEG | NR | NR | NR | NR | NR | 1Hz 50 Hz | 3 1 | 40 7 | 4 mA | 10 (100%) | | 90% Engel I,  10% Engel IV | 10% in HFES | mean of 16.8 m |
| Bancaud J, et al. Electroencephalography and Clin Neurophysiol. **1974**;37(3):275–82. | France | | P | 10 | 30 | 22.5 | IO | SEEG | 10 | 0 | 0 | 0 | 0 | 50Hz | 1 | 5 to 6 | 6 V | 10 (100%) | | NR | NR | NR |
| Halgren E, et al. Brain. **1978**;101(1):83–117. | USA | | R | 36 | 36 | 26 | EO | SEEG | 0 | 0 | 36 | 0 | 0 | 1Hz 30Hz | NR | 20 NR | 10 mA | 36 (100%) | | NR | NR | NR |
| Chassoux F, et al. Brain. **2000**;123(8):1733–51 | France | |  | 28 | 39 | 23 | EO | SEEG | 10 | 11 | 1 | 6 | 0 | 1Hz 50 Hz | NR 1 | NR 5 | 1-3 mA | 25 (89%) | | 64% Engel I,  7% II,  14% III and  14% IVB. | NR | mean of 6 m |
| Trebuchon A, et al. JNNP. **2020.** *In press* | France, Italy | | R | 346 | 51 | 25 | EO | SEEG | 123 | 34 | 154 | 0 | 35 | 1Hz 50Hz | 2 0.5-1 | NR 2-6 | 0.5-5 mA | 262 (75.3%) | | NR | NR | up to 24 m |
