# Supplementary material for: Electrical Stimulation-Induced Seizures and Breathing Dysfunction: A Systematic Review of New Insights Into the Epileptogenic and Symptomatogenic Zones
Source: Front Hum Neurosci. 2021 Jan 22;14:617061. doi: 10.3389/fnhum.2020.617061 (PMC7862564; doi:10.3389/fnhum.2020.617061)
Supplement: Supplementary file 2 [file Table_2.DOCX]

Supplementary Material

# Supplementary Data

Search strategy for Cortical stimulation to localize the seizure onset zone:

MEDLINE (OVID)

1. Electric Stimulation/
2. “cortical stimulation*” .ab,kf,ti.
3. “brain stimulation*” .ab,kf,ti.
4. “electrical stimulation*” .ab,kf,ti.
5. 1 or 2 or 3 or 4
6. Seizures/
7. “SOZ” .ab,kf,ti.
8. “seizure onset zone*” .ab,kf,ti.
9. “ictal onset zone” .ab,kf,ti.
10. “epileptogenic zone*” .ab,kf,ti.
11. “symptomatogenic zone*” .ab,kf,ti.
12. 6 or 7 or 8 or 9 or 10 or 11
13. 5 and 12
14. (electrostimulation* or electro-stimulation*).ab,kf,ti.
15. 5 or 14
16. 12 and 15

Search strategy for Cortical stimulation to induce seizure symptomatology including breathing disturbances:

MEDLINE (Ovid)

1. Electric Stimulation/ or Motor Cortex/ or Brain Mapping/ or Animals/ or Cerebral Cortex/ or Adult/ or Humans/ or "cortical stimulation".mp
2. Central/ or Apnea/
3. 1 and 2
4. Epilepsy/ or Drug Resistant Epilepsy/ or epilepsy.mp.
5. 3 and 4
